# Whole blood transcriptomics reveals the enrichment of neutrophil activation pathways during erythema nodosum leprosum reaction

**DOI:** 10.3389/fimmu.2024.1366125

**Published:** 2024-04-23

**Authors:** Thabatta Leal Silveira Andrezo Rosa, Thyago Leal-Calvo, Isabella Forasteiro Tavares, Mayara Abud Mendes, André Alves Dias, Meire Hellen dos Santos Piauy, Marcella Feitosa da Silva Barboza, Marylee Kapuscinski, Fabrício da Mota Ramalho Costa, Maria Angela M. Marques, Andrea de Faria F. Belone, Anna Maria Sales, Mariana de Andrea Hacker, Marcia de Berredo Pinho Moreira, John T. Belisle, Milton Ozório Moraes, Maria Cristina Vidal Pessolani, Veronica Schmitz

**Affiliations:** ^1^ Laboratório de Microbiologia Celular, Instituto Oswaldo Cruz, Fundação Oswaldo Cruz, Rio de Janeiro, Brazil; ^2^ Laboratório de Hanseníase, Instituto Oswaldo Cruz, Fundação Oswaldo Cruz, Rio de Janeiro, Brazil; ^3^ Department of Microbiology, Immunology and Pathology, Colorado State University, Fort Collins, CO, United States; ^4^ Divisão de Pesquisa e Ensino, Instituto Lauro de Souza Lima, Bauru, Brazil

**Keywords:** leprosy, erythema nodosum leprosum, neutrophils, blood transcriptomics, RNAseq, thalidomide

## Abstract

**Introduction:**

Patients with the multibacillary form of leprosy can develop reactional episodes of acute inflammation, known as erythema nodosum leprosum (ENL), which are characterized by the appearance of painful cutaneous nodules and systemic symptoms. Neutrophils have been recognized to play a role in the pathogenesis of ENL, and recent global transcriptomic analysis revealed neutrophil-related processes as a signature of ENL skin lesions.

**Methods:**

In this study, we expanded this analysis to the blood compartment, comparing whole blood transcriptomics of patients with non-reactional lepromatous leprosy at diagnosis (LL, n=7) and patients with ENL before administration of anti-reactional treatment (ENL, n=15). Furthermore, a follow-up study was performed with patients experiencing an ENL episode at the time of diagnosis and after 7 days of thalidomide treatment (THAL, n=10). Validation in an independent cohort (ENL=8; LL=7) was performed by RT-qPCR.

**Results:**

An enrichment of neutrophil activation and degranulation-related genes was observed in the ENL group, with the gene for the neutrophil activation marker *CD177* being the most enriched gene of ENL episode when compared to its expression in the LL group. A more pro-inflammatory transcriptome was also observed, with increased expression of genes related to innate immunity. Validation in an independent cohort indicated that *S100A8* expression could discriminate ENL from LL. Supernatants of blood cells stimulated *in vitro* with *Mycobacterium leprae* sonicate showed higher levels of CD177 compared to the level of untreated cells, indicating that the leprosy bacillus can activate neutrophils expressing CD177. Of note, suggestive higher CD177 protein levels were found in the sera of patients with severe/moderate ENL episodes when compared with patients with mild episodes and LL patients, highlighting CD177 as a potential systemic marker of ENL severity that deserves future confirmation. Furthermore, a follow-up study was performed with patients at the time of ENL diagnosis and after 7 days of thalidomide treatment (THAL, n=10). Enrichment of neutrophil pathways was sustained in the transcriptomic profile of patients undergoing treatment; however, important immune targets that might be relevant to the effect of thalidomide at a systemic level, particularly *NLRP6* and *IL5RA*, were revealed.

**Discussion:**

In conclusion, our study reinforces the key role played by neutrophils in ENL pathogenesis and shed lights on potential diagnostic candidates and novel therapeutic targets that could benefit patients with leprosy.

## 1 Introduction

The ancient disease leprosy is an ongoing global health issue, especially in countries such as India, Brazil, and Nepal, where its endemic status is constantly reinforced by the high numbers of new cases each year (1). This chronic infectious disease, caused by *Mycobacterium leprae* and *M. lepromatosis*, can manifest as skin and peripheral nerve lesions, which without treatment may develop into serious and irreversible deformities. The clinical manifestation of the disease is highly linked to the host immune response, resulting in a broad spectrum of clinical forms (2). The Ridley and Jopling classification, which considers histopathological aspects together with clinical, bacteriological, and immunological criteria, has defined the clinical manifestations from the least severe, contained form, known as the tuberculoid polar form (TT), to the most disseminated form, characterized by a high bacillary load, known as the lepromatous polar form (LL). Between these polar forms lie forms presenting graduations of symptoms, which are recognized as the borderline tuberculoid (BT), borderline borderline (BB), and borderline lepromatous (BL) forms (3).

The long-lasting course of leprosy can be interrupted by episodes of acute inflammatory responses, known as leprosy reactions. Leprosy reactions are linked to the high morbidity associated with the disease and the aggravation of neuropathic symptoms (4, 5). About 30–50% of multibacillary patients, represented by the BL and LL forms, develop erythema nodosum leprosum (ENL) (5), and it is more frequently observed during leprosy treatment with multidrug therapy (MDT). However, ENL can also occur before the start of MDT or even years after treatment conclusion (6–8). ENL is characterized by the appearance of new nodular inflammatory cutaneous lesions, which may occur with systemic symptoms, such as fever, arthralgia, myalgia, and malaise (4)

Management of ENL episodes involves the use of immunosuppressive drugs to control the exacerbated inflammatory response, often requiring long-term use of corticosteroid and/or thalidomide, causing serious side effects (5, 9). Thalidomide is extremely efficient in ameliorating ENL symptoms (10, 11); however, the drug teratogenic properties limit its use to men and women beyond reproductive age. Therefore, it has only been approved for use in a few countries, such as India and Brazil (12). Although it is believed that the efficiency of thalidomide is due to its immunomodulatory effects, its exact molecular targets in ENL are not yet fully understood. The definition of the inflammatory pathways inhibited by thalidomide in ENL could help improve therapy by the use of alternative drugs with less side effects.

Although the mechanisms that trigger the reaction and the crucial pathways linked to the inflammatory response are still elusive, neutrophils are recognized as key players during ENL (13, 14). Early histological studies revealed a rich neutrophilic infiltrate in cutaneous ENL lesions (15, 16). In recent years, alterations in neutrophil physiology are described to occur during ENL. These include increased neutrophil degranulation (17), the presence of neutrophil extracellular traps (NETs) in ENL skin lesions (18), and the association of CD64 expression in the circulating neutrophil population with the severity of the episode (19). Moreover, after 7 days of thalidomide treatment the neutrophilic infiltrate decreases in cutaneous ENL lesions (18–21), suggesting that limiting neutrophil participation during ENL is important for its remission, and that the effectiveness of the drug might be, in part, linked to this phenomenon.

Given the countless questions that still require attention regarding prevention, immunopathogenesis, and therapeutic interventions in ENL, high throughput techniques are a resourceful investigative tool that can be utilized to unveil novel targets and pathways of interest. At present, most transcriptomic studies in leprosy have focused on skin samples and have reported increased expression of neutrophil genes in the context of ENL (22–24). While cutaneous manifestations are central in ENL, the systemic alterations that occur are poorly explored and understood. While investigating blood cells expression profile in leprosy, most studies explore differences between polar forms (25, 26) and the sole transcriptomic study of peripheral blood mononucleated cells (PBMCs) from ENL patients strengthened the importance of inflammatory pathways (27). Nevertheless, the absence of neutrophils in this particular study limited the overall delineation of important pathways to the systemic inflammatory process.

A goal of the study reported here was to provide a whole blood transcriptomic profile of patients with active ENL and compare it with that of patients with non-reactional LL for a better understanding of the systemic pathogenic events associated with ENL. Secondly, whole blood transcriptomics analyses of patients with ENL before and after 7 days of thalidomide treatment was conducted as a strategy to identify thalidomide-modulated pathways, and thus, identify potential targets for efficient ENL treatment.

## 2 Methods

### 2.1 Patients

Patients with leprosy enrolled in this study were assisted at the Souza Araujo Outpatient Unit (Fundação Oswaldo Cruz, Rio de Janeiro, Brazil), a reference center for leprosy diagnosis and treatment. The study population was divided into two groups: 1) newly diagnosed lepromatous leprosy (LL) patients, treatment naïve, with no signs of reaction; and 2) patients with reaction diagnostics (ENL) before starting anti-reactional treatment, previously diagnosed with BL/LL forms of leprosy. Patients were enrolled in three main different assays: RNAseq {ENL (n=15), LL (n=7)}; RT-qPCR assay {ENL (n=8), LL (n=7)}; CD177 ELISA {ENL (n=28), LL (n=21)}. Patients with ENL were also followed up on the seventh day of thalidomide therapy.

Leprosy diagnosis was based on the presence of hypopigmented, anesthetic skin patches, thickened nerves, histopathological skin biopsies analysis, and acid-fast bacilli in skin smears (3). Patients with leprosy received MDT as recommended by the World Health Organization (WHO). ENL diagnosis was based on the detection of an acute appearance of crops of tender cutaneous or subcutaneous lesions with or without any systemic symptoms. Patients with ENL were enrolled in the longitudinal study when treated with thalidomide (100–300 mg daily) {RNAseq (n=10); RT-qPCR assay (n=8)}, in compliance with Brazilian Ministry of Health guidelines. Patients with ENL included in this study did not receive corticosteroids or immunomodulatory drugs at least 6 months before sample collection. Healthy donors (HD) were also included in the study for *in vitro* analysis (n=7). None of the patients or HD presented co-morbidities, such as co-infections, diabetes, or cancer.

The study was approved by the Instituto Oswaldo Cruz ethics committee, CAAE number 56113716.5.0000.5248.

### 2.2 Blood samples

Blood samples were collected after the informed consent form was signed by the participant. Peripheral whole blood samples were collected from all leprosy patients and HD enrolled in the study. A second point of collection was included in leprosy patients with ENL 7 days after starting thalidomide treatment. Approximately 2.5 mL of peripheral blood was collected in PAXgene Blood RNA Tubes (Qiagen, Germany) for transcriptomic analysis and quantitative reverse transcription polymerase chain reaction (RT-qPCR). In addition, blood samples were also collected in heparin-coated vacutainer collection tubes (BD, USA) and tubes with no additives (BD, USA), which were used, respectively, for whole blood culture or to obtain serum for CD177 protein quantification. Serum was obtained by incubating samples for 15 minutes at room temperature, followed by centrifugation at 300 *x g* for 10 min at 4°C for serum collection at the supernatant and storage at -80°C.

### 2.3 ENL severity

The clinical severity of the ENL cases was determined according to the ENL severity scale defined by ENLIST (12). This is a 10-item scale comprising: visual scale of pain; fever; the number, inflammation, and extent of ENL skin lesions; peripheral edema; bone pain; inflammation of joints and/or digits; lymphadenopathy; and nerve tenderness owing to ENL. Patients whose clinical records at the time of sample collection encapsulated the aforementioned information were categorized. Conversely, patients with incomplete records were disregarded from classification and were designated as “not classified.”

### 2.4 RNA extraction and sequencing

Whole blood transcriptomics were performed with blood samples collected in the PAXgene Blood RNA Tubes. Total RNA was obtained using the PAXgene Blood RNA kit (Qiagen, Germany) following the manufacturer’s protocol, including the kit’s DNAse treatment, as instructed. Agilent TapeStation 2200 (Agilent, USA) was used to assess RNA quality and all RNAs presented an RNA integrity number above 8. mRNA was enriched using the NEBNext Poly(A) mRNA Magnetic Isolation Module (New England Bioscience, USA). cDNA library preparation was performed using the NEBNext Ultra II Directional RNA Library Prep kit for Illumina (New England Bioscience, USA). The libraries were sequenced at the Next-generation sequencing core facility (Colorado State University, USA) using the NextSeq 500/550 High Output kit v2.5 (75 cycles) (Illumina, USA) on the Illumina NextSeq 550 platform. The raw data were deposited in Gene Expression Omnibus (GSE198609). **Supplementary Table S1** summarizes the baseline characteristics of the patients included in the RNAseq analysis.

### 2.5 RNAseq analysis

Initially, quality control (QC) of reads obtained after sequencing was accomplished using FastQC v.0.11.8 (https://www.bioinformatics.babraham.ac.uk/projects/fastqc/) and MultiQC v.1.9 (28), followed by removal of adapters using fastp v.0.21.0. In this step, poly(A) sequences were also removed, followed by trimming the initial 10 bases at the 5’-end. Sequence reads were mapped and quantified using the reference human transcriptome (GRCh38p.12, http://refgenomes.databio.org/v2/asset/fa159612d40b1bedea9a279eb24999b3d27145f9dd70dcca/salmon_index/splash?tag=default) according to the Salmon v.1.4. pipeline using the –gcBias and –seqBias flags, and transcript quantification was summarized into ENSEMBLE genes with tximport v.1.12.0 (29) and biomaRt v.2.40.5 (30). Differential gene expression (DGE) analysis using DESeq2 v.1.24.0 (31) was performed with ENL versus LL (adjusted for sex) groups, and paired samples of patients with ENL at day 7 of thalidomide treatment versus at the time of diagnosis (THAL vs. ENL). P-values were adjusted for multiple testing using the Benjamini and Hochberg procedure to control the false discovery rate (FDR) (32), and fold-changes (FC) were moderated with the “ashr” adaptive estimator (33). Genes considered differentially expressed complied with the following: |log_2_FC| ≥ 0.585 (FC ≥ 1.5) and FDR ≤ 0.1. The normalized expression matrix was transformed using shifted logarithm (base 2) and standardized to mean zero and unit standard deviation for the heatmap and unsupervised hierarchical clustering. Hierarchical clustering of genes and samples was done using the R package pheatmap v.1.0.12 with the Pearson correlation coefficient as the distance metric (34). Pathway enrichment analysis was performed using “biological processes” from Gene Ontology database through over-representation analysis (ORA) using the package cluterProfiler v.4.10.0 (35–38). All P-values reported are two-tailed. For principal component analysis (PCA), the top 500 most variable genes after normalization were used, applying DESeq2’s variance stabilization procedure (blinded concerning groups).

### 2.6 Quantitative reverse transcription PCR

Reverse transcription was performed on whole blood RNA using Superscript IV VILO Master Mix (Invitrogen-Thermo Fisher Scientific, USA) and RT-qPCR reactions were performed in duplicates on a ViiA7 Real-Time PCR System (Applied Biosystems, USA) using PowerUp SYBR Green Master Mix (Applied Biosystems-Thermo Fisher Scientific, USA). For reactions, 10 ng of cDNA and 400 nM of oligonucleotide primers were used (except for *CD177* primers, which were used at 800 nM). The sequences of primers are available in **Supplementary Table S2**. The genes *RPL13a* and *RPS16* were used as reference genes for normalization. The relative gene expression analysis was performed using the efficiency-adjusted N0 method as previously described (39, 40). Briefly, raw data were exported from ViiA7 proprietary software and imported into LinRegPCR v.2022 for obtaining N0 values. The N0 values were normalized by taking their ratio to the normalization factor calculated using the geometric mean of N0 values for the reference genes. Normalized expression values were converted into log_2_ before statistical analyses and visualization in R v.4.1. Statistical significance of the difference between the two means was assessed using Welch’s t-test, while the paired t-test was used for the follow-up design. Additionally, Spearman’s correlation tests were performed between *CD177* gene expression levels and the remaining subset of genes analyzed in patient samples of the ENL group using GraphPad Prism v.8.0.0.

### 2.7 Whole blood cell culture with *M. leprae* sonicate

We collected two milliliters of whole blood from a group of seven individuals, consisting of two males and five females, with an average age of 31 years (referred to as “HD”). The blood was placed directly into 24-well plates with no additional medium, and then incubated at 37°C and 5% CO_2_ for 24 hours in the presence or absence of *M. leprae* sonicate (NR19329, BEI Resources, USA) at a concentration of 10 μg/mL. Following incubation, the whole blood samples were subjected to the previously described centrifugation process (17), and the plasma was analyzed for CD177 measurement.

### 2.8 CD177 protein measurement

Concentrations of CD177 protein in the serum samples of patients (diluted 1:2) or plasma of whole blood cell cultures (diluted 1:4) were determined via a commercial enzyme-linked immunosorbent assay kit (ELISA), according to the manufacturer’s protocol (Cat#: EH80RB, Invitrogen-Thermo Fisher Scientific, USA). Readings were performed in the EON Microplate spectrophotometer (BioTek, USA) and data were analyzed using Gen5 software (BioTek, USA). Mean absorbances of duplicate readings for each sample were compared with those of the standard curve to give the concentrations, given in ng/mL. The minimum detectable concentration of human CD177 was 0.21 ng/mL.

### 2.9 Statistical analysis

Statistical analysis of CD177 levels was carried out using GraphPad Prism v.8.0.0. For the analysis of the plasma levels after whole blood cell stimulation with *M. leprae* sonicate, a two-tailed paired t-test was performed after the normal distribution was confirmed by the Shapiro–Wilk test. For analysis of the patient serum levels, normal distribution was first determined using the Shapiro–Wilk test then the two-tailed Welch’s t-test was applied. For all statistical analyses, a P-value was considered statistically significant when lower than 0.05.

## 3 Results

### 3.1 Patient clinical background

Characteristics of the patients with leprosy enrolled in this study are shown in **Supplementary Tables S1, S3, S4**. Patients were divided into either treatment-naïve with the LL form without any sign of reaction at leprosy diagnosis (LL group) or those with active ENL before starting anti-reactional treatment (ENL group). The median age of the LL group was 38 years (interquartile range, 30–51), and 70% were men. The ENL group comprised 75% of men and the median age was 40 years (interquartile range, 31–55). None of the patients of the LL group developed reaction during the first 4 months of MDT treatment. Blood samples of all patients were collected for analysis, and a second sampling was performed for the patients of the ENL group after 7 days of thalidomide treatment (THAL group).

### 3.2 Genes associated with neutrophil activation and degranulation are enriched in the blood transcriptomics of patients with ENL

**Figure 1:**
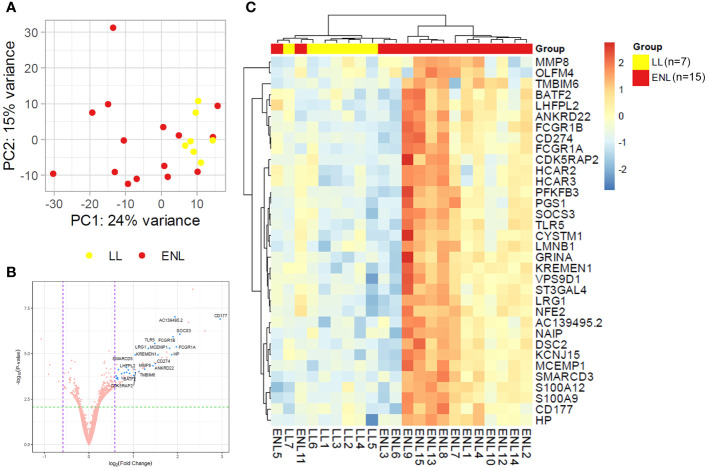
Patients with lepromatous leprosy experiencing ENL display a distinct blood transcriptomic profile to that of non-reactional patients. **(A)** Principal component analysis of the 500 most variable genes in patients with lepromatous leprosy and active erythema nodosum leprosum (ENL; red, n=15) compared to those that were non-reactional (LL; yellow, n=7). **(B)** Volcano plot analysis of differentially expressed genes in ENL vs. LL. Differentially expressed genes are depicted in blue. **(C)** Heatmap showing the differentially expressed genes in the blood of ENL patients over LL patients. Expression levels are represented by a scale of log_2_FC from -3 (dark blue) to 3 (bright red).

Genome-wide transcriptional profiles were generated from the blood of patients from ENL group (n = 15) versus patients from the LL group (n = 7). A dataset of 16,525 expressed genes was generated, and the PCA of the 500 most differentially expressed genes was unable to separate samples into two distinct groups (**Figure 1A**). Although LL patient samples were tightly grouped, ENL patient samples were scattered between the two first components, highlighting their greater heterogeneity. Thirty-four statistically significant differentially expressed genes were identified, all of which were enriched in ENL compared to their levels in LL (**Figure 1B**; **Supplementary Table S5**). The neutrophil activation marker *CD177* (41) was the most enriched gene in ENL, increased by about 8-fold compared to the level in LL (log_2_FC = 2.96, adj. p = 0.0003), followed by *SOCS3* (log_2_FC = 2.05, adj. p = 0.001), a master regulator of the immune response (42), and *FCGR1A* (log_2_FC = 1.97, adj. p = 0.003) and *FCGR1B* (log_2_FC = 1.90, adj. p = 0.003), encoding the Fc gamma receptors 1A (CD64A) and 1B (CD64B), respectively (43, 44). A heatmap comprising all 34 differentially expressed genes was drawn (**Figure 1C**). A homogeneous profile was observed for LL patients in agreement with the PCA pattern. In contrast, ENL patients clearly showed a more heterogeneous profile, with a few (n = 4) presenting an expression profile more similar to that of LL patients, aligned with the diverse symptomatology observed clinically. In fact, the categorization of severity of the ENL episodes according to the ENLIST criteria exhibited significant heterogeneity (**Supplementary Table S1**). However, a tentative to correlate the RNAseq profile with the severity and/or chronicity of the reaction failed, with no identification of discernible patterns according to the ENLIST severity status.

**Figure 2:**
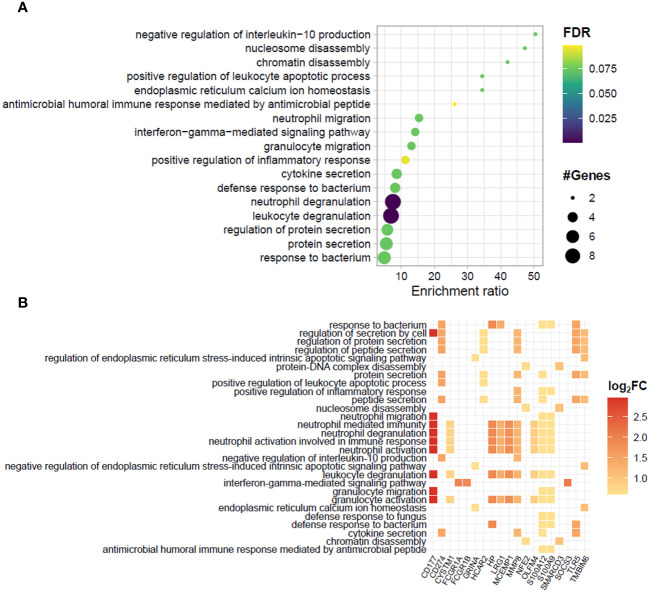
Biological processes and related genes enriched in the blood transcriptomics of patients experiencing ENL. **(A)** Dotplot and **(B)** heatplot of enriched biological processes associated with positively regulated genes in patients with erythema nodosum leprosum (ENL) obtained by the over-representation analysis (ORA). In **(A)**, pathways are depicted in a hierarchy according to the enrichment ratio. Circle size and color indicate, respectively, the number of genes associated with the pathway and the P-value according to the false discovery rate (FDR). In **(B)**, the identity of the genes associated to each enriched biological process is given. The square color indicates the log_2_FC value according to the scale.

A functional analysis of the genes significantly enriched in the blood of ENL patients by over-representation analysis (ORA) showed an abundance of genes involved in innate immunity and inflammatory pathways (**Figures 2A, B**). Notably, 9 out of the 34 upregulated genes (26.5%) were associated with neutrophil/granulocyte activation, degranulation, and migration pathways (**Figure 2B**). These genes were *CD177, CYSTM1, HP, LRG1, MCEMP1, MMP8, OLFM4, S100A12*, and *S100A9*. This was an expected observation based on previous literature reports of neutrophilia during ENL (45–47). Indeed, we also observed higher neutrophil counts in the blood of a subset of ENL patients from the RNAseq cohort with available blood counts by the time they were recruited for the study (LL= 7; ENL = 5) (**Supplementary Figure S1**). Other pathways with enrichment included “response to bacterium” with 5 associated genes including *TLR5*, and “interferon-gamma-mediated signaling pathway” with 3 associated genes, *FCGR1A, FCGR1B, and SOCS3*.

**Figure 3:**
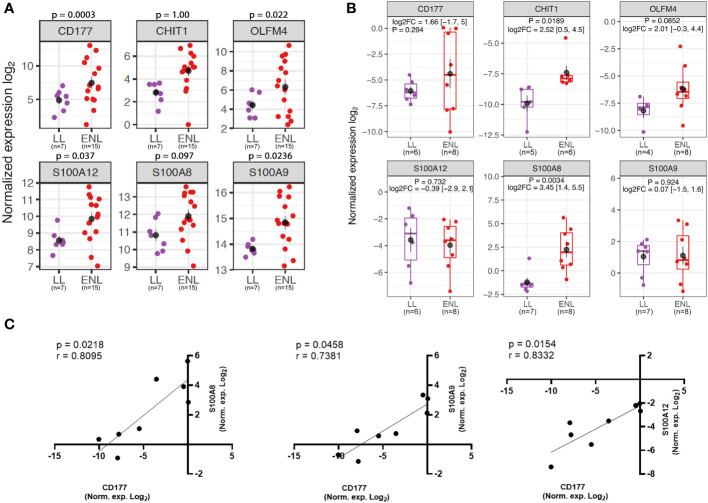
Neutrophil genes are enriched in the blood transcriptomics of patients experiencing ENL. **(A)** Normalized expression levels from RNAseq differential gene expression (DGE) analysis of patients with lepromatous leprosy who were non-reactional (LL; purple, n=7) or had active erythema nodosum leprosum (ENL; red, n=15) for neutrophil genes: *CD177*, *CHIT1*, *OLFM4*, *S100A12*, *S100A8*, and *S100A9*. **(B)** Log normalized expression levels of the neutrophil genes *CD177* (LL=6; ENL=8), *CHIT1* (LL=5; ENL=6), *OLFM4* (LL=4; ENL=8), *S100A12* (LL=6; ENL=8); *S100A8* (LL=7; ENL=8) and *S100A9* (LL=7; ENL=8) obtained by RT-qPCR in a new cohort of LL (purple) and ENL patients (red). The genes *RPL13* and *RPS16* were used as reference controls for gene expression normalization. Statistical analysis was performed by applying Welch’s t-test. **(C)** Spearman’s correlation test of normalized expression log_2_ data from RT-qPCR of ENL patients between *CD177* and *S100A8* (left panel), *CD177* and *S100A9* (middle panel), and *CD177* and *S100A12* (right panel). Positive correlation and significance are considered when the r-value is > 0.7 and the p-value is < 0.05, respectively.

To further explore neutrophil associated genes, the genes *CD177*, *CHIT1*, *OLFM4*, *S100A12*, *S100A9*, and *S100A8* were selected based on their extensive use as markers of neutrophilic processes (48–53). Of note, the inclusion of the genes *S100A8* and *CHIT1* in this analysis, despite their absence from differentially expressed genes, was based on the intimate relation between *S100A8* and *S100A9*, which often act as a heterodimer (54), and the presence of *CHIT1* among top positively regulated genes in the subsequent longitudinal study. The expression levels of neutrophil-related genes in the RNAseq for each patient showed an overall heterogeneous profile within the ENL group and some patients had markedly increased expression compared to the levels in non-reactional LL patients (**Figure 3A**). A similar pattern of enrichment was also observed with the *CHIT1* and *S100A8* genes that, although not among the differentially expressed genes from the RNAseq data, had higher average expression levels in ENL patients compared to the levels in LL patients (**Figure 3A**). Replication of RNAseq results by RT-qPCR in an independent patient cohort (LL=7; ENL=8) revealed a similar distribution pattern of the expression levels for the chosen neutrophil genes (**Figure 3B**). Although the mean expression values obtained by RT-qPCR were higher in ENL patients than in LL patients for four genes among six tested, a significant difference was only observed for *S100A8* and *CHIT1* expression. Nevertheless, a positive correlation was observed between the normalized expression levels of *CD177* and all three S100 genes analyzed in ENL patients (**Figure 3C**), where the strongest correlation was observed with *S100A12* (r = 0.8332; p = 0.0154), followed respectively by correlation with *S100A8* (r = 0.8095; p = 0.0218) and *S100A9* expression (r = 0.7381; p = 0.0458), while no correlation was observed with the remaining set of genes (data not shown).

**Figure 4:**
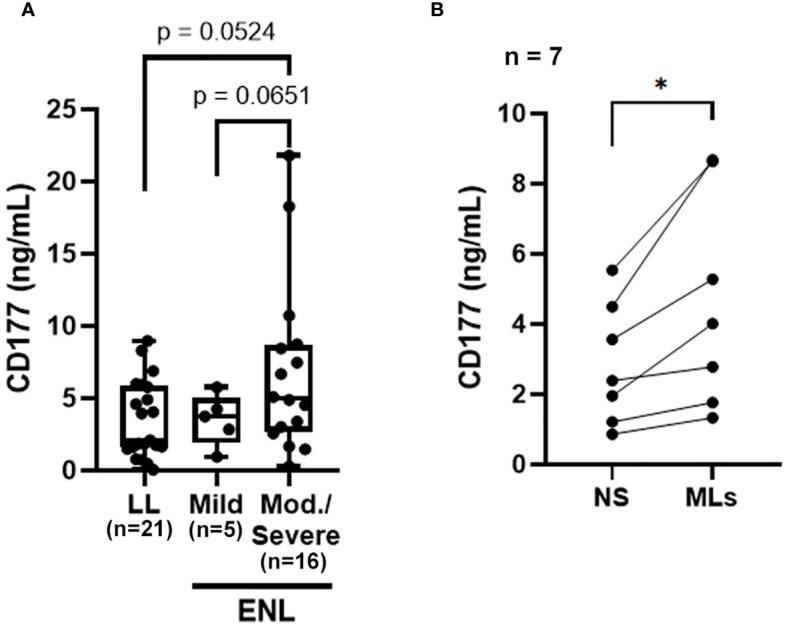
The CD177 molecule may be related to ENL severity. **(A)** CD177 levels in sera from non-reactional lepromatous leprosy (LL; n=21) and ENL patients classified according to the ENLIST ENL Severity Scale: mild ENL reaction (n=5) and moderate-severe reaction (n=16). Statistical analysis was performed using a two-tailed Welch’s t-test. **(B)** Whole blood cells from healthy donors (n=7) were stimulated with 10 µg/mL of *M. leprae* sonicate (MLs) or not (NS, non-stimulated) for 24 h and the CD177 protein levels in the plasma were determined by ELISA. Statistical analysis was performed using a two-tailed paired t-test (*p<0.05).

A suggestive increase in protein levels of CD177 was found in serum samples of ENL patients with the median value about twice that of the levels in LL patients (ENL median: 4.016 ng/mL; LL median: 2.094 ng/mL, p = 0.1210) (**Supplementary Figure S2**). Notably, subsequent ENL severity stratification according to ENLIST parameters revealed suggestive higher serum CD177 levels in patients with moderate or severe episodes compared to those of patients with mild episodes (p = 0.0651), as well as comparing with LL patients (p = 0.0524) (**Figure 4A**). Moreover, *in vitro* stimulation of whole blood cells from HD with *M. leprae* sonicate significantly increased the release of this protein into the plasma (**Figure 4B**), suggesting that mycobacterial components may activate neutrophils expressing CD177 *in vivo*. Taken together, these results reinforce the key role played by neutrophils in ENL immunopathogenesis and suggest that the severity of the reactional episode may be linked to the degree of neutrophil activation.

### 3.3 Effect of thalidomide treatment on the blood transcriptomics of patients with ENL

**Figure 5:**
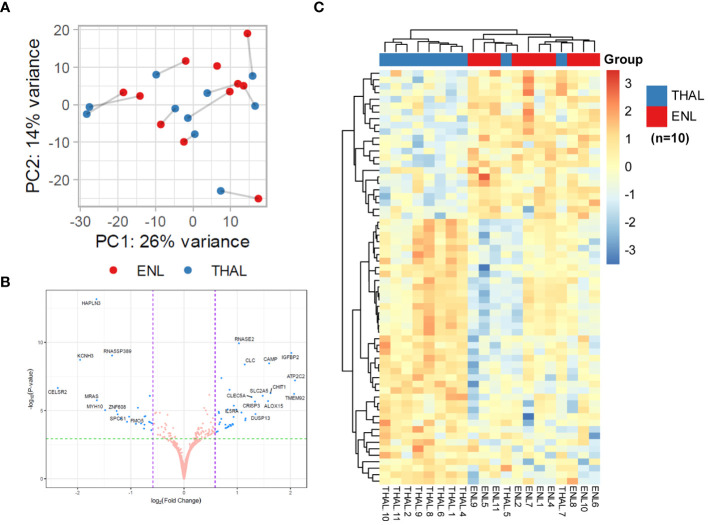
Pairwise comparisons for patients with ENL before and after 7 days of thalidomide treatment to determine transcriptional changes in the blood. **(A)** Principal component analysis of the 500 most variable genes in patients with erythema nodosum leprosum (n=10) at diagnosis (ENL; red) and after 7 days of thalidomide treatment (THAL; blue). **(B)** Volcano plot analysis of differentially expressed genes in the ENL and THAL groups. Significantly expressed genes are represented in blue. **(C)** Heatmap of differentially expressed genes in the blood cells of the ENL (red) and THAL (blue) groups. The scale on the right represents the expression levels of each gene ranging from a log_2_FC value of -4 (dark blue) to 4 (bright red).

Together with establishing a blood transcriptomic profile of patients experiencing ENL, this study sought to determine the profile of these patients after starting thalidomide treatment (THAL). A segment of the patients with ENL who were administered thalidomide therapy and met the specified criteria delineated in the methodology section were recruited for participation in the longitudinal investigation (n=10). The timepoint of choice for sample collection was at the seventh day of treatment, the time at which several indications of treatment efficacy have previously been reported, especially for cutaneous symptoms (10, 11). Owing to the heterogeneous pattern of gene expression among patients with ENL, pairwise comparisons for each patient were performed to determine the transcriptional changes. As expected, the PCA of the 500 most variable genes was unable to separate samples into two distinct groups highlighting their heterogeneity (**Figure 5A**). In this RNAseq analysis, 16,527 transcripts were detected. However, only 64 genes were found to be significantly differentially expressed (**Figure 5B**). Hierarchical clustering shows less prominent regulation of these 64 genes after 7 days of treatment in two patients, presenting a transcriptional profile more similar to patients before treatment. The hierarchical clustering also showed a more homogeneous gene expression pattern after thalidomide treatment in comparison with that before treatment, with the upper third of the heatmap displaying the downregulated genes, and the lower two-thirds displaying the upregulated genes in the THAL samples (**Figure 5C**).

DGE analysis of patients with ENL before and after thalidomide treatment found a total of 41 positively regulated genes and 23 negatively regulated genes in the THAL group when compared to the levels in the ENL group (**Supplementary Table S6**). The three most upregulated genes were *ATP2C2* (log_2_FC = 2.09, adj. p < 0.001), *TMEM92* (log_2_FC = 2.07, adj. p < 0.001), and *IGFB2* (log_2_FC = 2.02, adj. p < 0.001). Among the upregulated genes, some immunity-related genes were observed, such as *CAMP* and *IL5RA* (**Figure 5B**; **Supplementary Table S6**).

**Figure 6:**
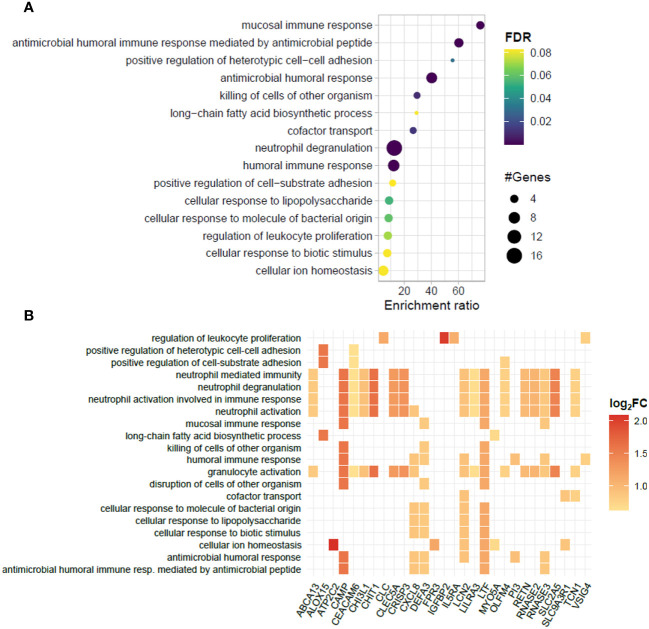
Biological processes represented in the blood transcriptomic profiles of patients with ENL before and after 7 days of thalidomide treatment. **(A)** Dotplot portraying the enriched pathways in blood cells of patients with erythema nodosum leprosum (ENL) at day 7 of thalidomide treatment (THAL group), compared to the moment of ENL diagnosis (ENL group). The pathways are hierarchically positioned according to the enrichment ratio. Circle size indicates the number of genes associated with the pathway and the color indicates the P-value according to the false discovery rate (FDR). **(B)** Heatplot of enriched biological processes associated with genes positively regulated in the THAL group obtained by over-representation analysis (ORA). Values of log_2_FC are color-graded according to the scale.

Although no significantly enriched pathways were found for the set of downregulated genes, some of these genes are associated with important immune regulation processes, such as the inflammasome-related gene *NLRP6*, and *MRAS*, which encodes a regulatory protein from the RAS family GTPases (**Supplementary Table S6**). For the upregulated genes, over 20 enriched pathways were found; however, 41% of the genes (17 out of 41) were still associated with neutrophil activation/degranulation pathways, suggesting the persistence of neutrophil activation despite the initiation of thalidomide treatment (**Figures 6A, B**). Besides the pathways linked to neutrophil activation, several unique biological processes, unseen in the ENL vs. LL transcriptomic analysis, were found enriched during thalidomide treatment, such as “humoral immune response” and “antimicrobial humoral response” with 8 genes each, and “mucosal immune response” and “antibacterial humoral immune response mediated by antimicrobial peptide” with 4 genes each (**Figures 6A, B**).

**Figure 7:**
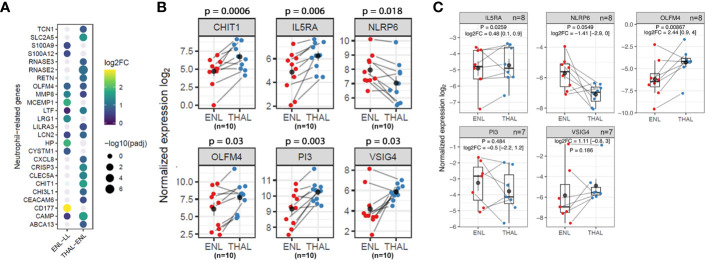
Neutrophil genes and pathways are still overrepresented in thalidomide-treated patients with ENL. **(A)** Dotplot representing the neutrophil genes differentially expressed in the ENL vs. LL comparison and in the THAL vs. ENL paired comparison. The circle size represents the log of the adjusted p-values. The circle color represents the log_2_FC values according to the scale. **(B)** Normalized expression levels from RNAseq differential gene expression (DGE) analysis of the longitudinal analysis of patients with ENL (n=10) at diagnosis (ENL group; red) and after 7 days of thalidomide treatment (THAL group; blue) for *CHIT1*, *OLFM4*, *IL5RA*, *PI3*, *NLRP6*, and *VSIG4* genes **(C)** Expression levels of *IL5RA* (n=8), *PI3* (n=7), *NLRP6* (n=8), *VSIG4* (n=7) and *OLFM4* (n=8) genes obtained by RT-qPCR in a new cohort of patients with ENL at diagnosis (ENL; red) and after 7 days of thalidomide treatment (THAL; blue). The genes *RPL13* and *RPS16* were used as reference controls for gene expression normalization. Statistical analysis was performed using a paired t-test.

The majority of upregulated genes associated with neutrophilic pathways during thalidomide treatment, including *CAMP*, *CHIT1*, *LTF*, and *LCN2* genes, are broadly different from those observed in the ENL vs. LL comparison (**Figure 7A**) and are strongly associated with neutrophil granules (55, 56). This sustained neutrophil participation even during thalidomide treatment can be inferred not only by the pattern of increased expression levels of the neutrophilic markers, *CHIT1* and *OLFM4*, in the RNAseq analysis (**Figure 7B**), but also by the increased *OLFM4* expression in the RT-qPCR replication analysis using a different patient cohort (**Figure 7C**).

A detailed inspection of the positively regulated genes revealed interesting targets that might contribute to subsiding the inflammatory process during an ENL episode, such as the genes *VSIG4*, *PI3*, and *IL5RA* (**Figure 6B**). Indeed, the pairwise comparison of the expression levels of these genes revealed higher levels after thalidomide treatment in most patients (**Figure 7B**). The RT-qPCR replication analysis identified the same expression pattern for *IL5RA* (log_2_FC = 0.48; p = 0.0259). The overall gene expression behavior of *VSIG4* by RT-qPCR was also like that seen in the RNAseq. While the difference between *VSIG4* averages was not statistically different, the higher levels of expression during thalidomide treatment may be worth future investigation with larger cohort of samples. In contrast, RT-qPCR analysis of *PI3* showed high heterogeneity, underpowering the experiment to detect average differences (**Figure 7C**). *NLRP6* was also selected for analysis because of its association with the inflammasome. In most patients, the expression levels of *NLRP6* were reduced by thalidomide treatment in the RNAseq analysis (**Figure 7B**). A similar result was observed in the RT-qPCR analysis with a distinct cohort of patients (log_2_FC = -1.41; p = 0.0549) (**Figure 7C**), confirming the differences observed in the RNAseq.

## 4 Discussion

Whole blood transcriptomic analysis as a tool to better understand diseases has been increasingly used over the last decade (57, 58). Our study applied this approach to analyze patients with active ENL, as well as thalidomide-induced changes in ENL patients under treatment to better understand the pathways important for ENL management. Our RNAseq data demonstrated a highly inflammatory profile in the blood of patients with active ENL compared to non-reactional lepromatous patients. The most prominent enriched genes in ENL patients were those associated with neutrophil activation and degranulation. This was an expected result based on the neutrophilia previously described and herein confirmed in ENL patients, and the increased levels of activation and degranulation already reported in circulating ENL neutrophils (13, 14, 17–20). The data obtained in blood align with the neutrophilic infiltrate in ENL skin lesions (15, 16), and the recent meta-analysis of available transcriptomic data of skin lesions highlighting the upregulation of pathways associated with a neutrophil response in ENL (24). A neutrophil-enriched blood transcriptomic profile was described in tuberculosis (59), rheumatoid arthritis and SLE (60–62). Interestingly, the neutrophilic signature in SLE has been linked to an increased risk of vascular inflammation (63), which can also be observed in a portion of ENL skin lesions (64). In conclusion, our findings strengthen the importance of neutrophils during ENL, suggesting that they are a linchpin of ENL immunopathogenesis.

Although PBMC preparations are devoid of the normal-density neutrophils, Dupnik et al. (2015) reported neutrophil-related genes among the ones upregulated in the PBMCs of ENL patients (27), such as *OLFM4*, *LTF*, and *LCN2*. This observation could have been related to an increased low-density neutrophil population reported in ENL patients (17). A major hypothesis formulated by the authors was that low-density neutrophils are formed because of degranulation, which would indicate the augmented degranulation and activation in the neutrophil population (17). Our study corroborates this previous finding by not only demonstrating a neutrophilic signature profile in blood cells of patients with ENL, but also by observing the enrichment of the biological process from gene ontology of “neutrophil degranulation”.

In our RNA-seq analysis, *CD177* appeared as the most differently expressed gene in ENL when compared to non-reactional LL patients. CD177 is commonly acknowledged as a marker of neutrophil activation (65, 66) and it has been associated with neutrophil migration (67), degranulation (68) and NET formation (69) Increased numbers of circulating CD177^+^ neutrophils have also been reported in other systemic inflammatory diseases, such as sepsis (70), ANCA-associated systemic vasculitis (AAV), and SLE (71). Of note, *CD177* expression levels in our study were very heterogeneous. This observation was not unexpected as *CD177* variability in the human population is markedly linked to an individual’s genetic background (72). We found suggestive increased CD177 serum levels in moderate-severe reactional episodes compared to those found in mild episodes, pointing CD177 as a potential biomarker candidate for ENL severity, which deserves confirmation in future studies. Interestingly, CD177 has also been recently linked to COVID-19 severity (48, 65, 73).

Moreover, several genes identified in our study with higher expression levels in whole blood during the active episode of ENL corroborate previous studies. Notably, this includes genes such as *S100A8*, *S100A9*, and *S100A12*, whose respective encoded proteins are reported to be elevated in the serum of ENL patients (74, 75). The S100 protein family is intimately linked to neutrophil activation (54, 76) and inflammatory processes, and diseases such as sepsis, rheumatoid arthritis, psoriasis and SLE (54, 77). Moreover, S100A8 and S100A9 can stimulate transendothelial migration of neutrophils into affected tissue (78–80) and recently were defined as markers of NETosis (76). Increased expression of *S100A8/S100A9* was also observed in ENL skin lesions (22), reinforcing their role as key players of ENL inflammation. Remarkably, in the RT-qPCR cohort, we also observed a positive correlation of expression of *CD177* with those of *S100A8/A9/A12*, where the pattern of increased expression level of one gene matches the other, suggesting an interplay of these neutrophil-associated genes during ENL. Interestingly, several studies have observed a combined increased expression of these genes in different inflammatory contexts, such as sepsis (81) and COVID-19 (82), strengthening the correlation among these genes. Nevertheless, the precise mechanistic role of S100 proteins during ENL requires further study.

The increased expression of *FCGR1A* and *FCGR1B*, which code for distinct forms of the high affinity FCγ receptor (CD64), in ENL patients agrees with previous descriptions of increased CD64 on both circulating neutrophils (19) and PBMCs from ENL patients (27). Moreover, increased levels of CD64^+^ neutrophils have been associated with the severity of the ENL episode (19). The *FCGR1B* gene is induced in monocytes and neutrophils by IFN-γ (83). The upregulation of *FCGR1B* expression is, therefore, substantiated by the extensive number of studies reporting the IFN-γ contribution during ENL, such as the observations of increased IFN-γ levels in patient sera (84, 85), overexpression of IFN-γ and related genes in PBMCs (27, 86, 87), and in ENL skin lesions (22, 86).

Several reports demonstrate the capacity of thalidomide to modulate aspects of neutrophil biology (88), such as decreasing neutrophil phagocytosis (89), transendothelial migration to sites of inflammation (90, 91), and downregulation of the neutrophil activation marker CD11b (92). Previous studies on ENL report a reduction of the neutrophilic inflammatory infiltrate (18–20) and NET formation (18) in ENL skin lesions after 7 days of thalidomide treatment. However, our blood transcriptomics analysis revealed the persistence of inflammatory pathways, including those linked to neutrophil activation and degranulation, at day 7 of treatment with thalidomide. A prominent neutrophil gene during thalidomide treatment was *CHIT1.* Augmented gene expression of *CHIT1* was reported in ENL skin lesions (93) and increased chitotriosidase activity in the serum of ENL patients in comparison to those of healthy individuals have been reported (94). *CHIT1* encodes the enzyme chitotriosidase (or chitinase 1), which is linked to neutrophil granules (95) and associated with an intense inflammatory profile (96–98). Altogether, these data indicate that a longer period of treatment with the drug is required to observe reduction of neutrophil enrichment and activation systemically.

Despite the persistence of an inflammatory profile in the blood after a short period of thalidomide administration, several new upregulated genes/pathways with anti-inflammatory effects were detected. These include the gene V-set immunoglobulin-domain-containing 4 (*VSIG4*), that codes for a membrane protein belonging to complement receptor of the immunoglobulin superfamily (CRig). VSIG4 has been implicated in NLRP3 inflammasome inhibition (99), suppression of T cell activity (100) and inhibition of LPS-induced pro-inflammatory macrophage activity (101). Interestingly, successful administration of VSIG4 results in remission of rheumatoid arthritis (102) and decreases kidney deposition of immunocomplexes to ameliorate SLE-associated glomerulonephritis (103). This points to VSIG4 as a potential candidate for ENL therapy. The study of *VSIG4* upregulation in ENL patients under thalidomide treatment needs further confirmation with a larger cohort of samples and with longer periods of treatment.

Another important change in the transcriptomic profile observed during thalidomide treatment was the appearance of genes associated with humoral and Th2 immunity, such as *IL5RA*. This is in line with the observation that thalidomide treatment of PBMCs *in vitro* stimulates the production of Th2-related cytokines (104). Based on these data, we posit that the emergence of a Th2 response during thalidomide treatment counteracts the involvement of a T-cell mediated response in ENL pathology (105).

*NLRP6*, a gene that codes for the cytoplasmic receptor of the NLRP6 inflammasome, was also downregulated after thalidomide treatment. This gene is also linked to neutrophil migration and induction of NETosis during sepsis (106). Thus, based on this, we speculate that NLRP6 inflammasome participates in ENL pathogenesis and represents a potential inflammatory pathway targeted by thalidomide.

It is noteworthy to discuss the lack of modulation of genes directly associated with the TNF pathway, which is tightly associated with the action of thalidomide and its analogs as inhibitors of this cytokine (21, 107–111). In fact, our data are in line with a previous study where ENL patients were followed up during 21 days of thalidomide treatment. In that study, a modest increase in TNF serum levels was observed during treatment, even with evident improvement of skin lesions. These data together with observations of increased circulating levels of IL-12 and IL-2 and IFN-γ expression by T cells, suggests that thalidomide promotes a complex immunoregulatory response (112).

Finally, it is important to address the limitations of our study, which includes a modest sample size and intrinsic sex bias in the longitudinal study, due to restrictions of thalidomide administration to women within reproductive age. Moreover, the experimental design did not account for possible implications of MDT treatment in the transcriptional profile or CD177 serum levels of patients with ENL, although it was not found a clear distinguishable pattern among ENL patients that could be linked to different MDT status. Yet, further studies are advised to overcome these limitations and expand the current findings.

In summary, despite the limitations, our data, together with published reports, support the protagonist role of neutrophils during ENL immunopathogenesis and highlight their participation in a systemic context. Although immunomodulation by thalidomide was found to be modest in the blood compartment after a short period of treatment in patients with ENL, the expression of genes with potential anti-inflammatory roles was detected. This suggests a systemic path to the resolution of ENL associated inflammation. Our data also revealed potential diagnostic markers and new targets for therapeutic intervention for ENL management.

## Data availability statement

The datasets presented in this study can be found in online repositories. The names of the repository/repositories and accession number(s) can be found below: https://www.ncbi.nlm.nih.gov/geo/, GSE198609.

## Ethics statement

The studies involving humans were approved by Instituto Oswaldo Cruz ethics committee (CEP Fiocruz/IOC). The studies were conducted in accordance with the local legislation and institutional requirements. The participants provided their written informed consent to participate in this study.

## Author contributions

TR: Writing – original draft, Methodology, Investigation, Formal analysis. TL-C: Writing – original draft, Methodology, Investigation, Formal analysis. IT: Writing – review & editing, Methodology, Investigation. MAM: Writing – review & editing, Investigation, Formal analysis. AD: Writing – review & editing, Methodology, Investigation. MS: Writing – review & editing, Methodology, Investigation. MF: Writing – review & editing, Investigation, Formal analysis. MK: Writing – review & editing, Investigation. FC: Writing – review & editing, Methodology, Investigation. MMM: Writing – review & editing, Resources, Project administration, Funding acquisition. AF: Writing – review & editing, Resources. AS: Writing – review & editing, Investigation, Formal analysis. MH: Writing – review & editing, Formal analysis. MBP: Writing – review & editing, Supervision, Project administration. JB: Writing – review & editing, Supervision, Resources, Funding acquisition. MOM: Writing – review & editing, Supervision, Funding acquisition. MCVP: Writing – review & editing, Supervision, Resources, Funding acquisition, Conceptualization. VS: Project administration, Writing – review & editing, Supervision, Resources, Funding acquisition, Conceptualization.
