# Supplementary material for: Whole blood transcriptomics reveals the enrichment of neutrophil activation pathways during erythema nodosum leprosum reaction
Source: Front Immunol. 2024 Apr 23;15:1366125. doi: 10.3389/fimmu.2024.1366125 (PMC11074352; doi:10.3389/fimmu.2024.1366125)
Supplement: Supplementary file 1 [file DataSheet_1.docx]

**Whole blood transcriptomics reveals the enrichment of neutrophil activation pathways during erythema nodosum leprosum reaction**

**Thabatta Leal Silveira Andrezo Rosa^1†^, Thyago Leal-Calvo^2†^, Isabella Forasteiro Tavares^2^, Mayara Abud Mendes^2^, André Alves Dias^1^, Meire Hellen dos Santos Piauy^1^, Marcella Feitosa da Silva Barboza^2^, Marylee Kapuscinski^3^, Fabrício da Mota Ramalho Costa^3^, Maria Angela M. Marques^3^, Andrea de Faria F. Belone^4^, Anna Maria Sales^2^, Mariana de Andrea Hacker^2^, Marcia de Berredo Pinho Moreira^1^, John T. Belisle^3^_,_ Milton Ozório Moraes^2§^, Maria Cristina Vidal Pessolani^1#^ and Veronica Schmitz^2#*^**

Supplementary Figures


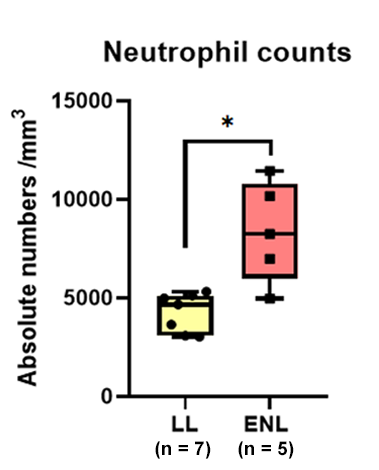


**Supplementary Figure S1.** **Neutrophil absolute numbers are increased in patients with ENL.** Blood counts available from patients with leprosy were assessed to determine neutrophil absolute numbers/mm^3^ in the cohort of RNAseq analysis (LL, n= 7; ENL, n= 5). Statistical analysis was performed using a two-tailed Welch’s t-test (* p < 0.05).


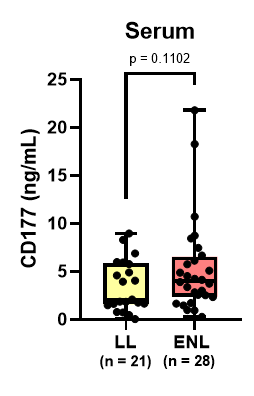


**Supplementary Figure S2.** **CD177 median levels in sera are increased in patients with ENL.** CD177 protein levels were determined in the sera of patients with non-reactional lepromatous leprosy (LL; n=21) or with active erythema nodosum leprosum (ENL; n=28). Results are shown as a boxplot with the median and interquartile range with minimum and maximum values, where each dot represents an individual patient. Statistical analysis was performed using a two-tailed Welch’s t-test.
